# Supplementary material for: Modeling glioblastoma heterogeneity as a dynamic network of cell states
Source: Mol Syst Biol. 2021 Sep 16;17(9):e10105. doi: 10.15252/msb.202010105 (PMC8444284; doi:10.15252/msb.202010105)
Supplement: Supplementary file 5 — Source Data for Figure 3 [file MSB-17-e10105-s001.zip › Figure3A_sourcedata/GSEA_3065/hallmarks_state4.GseaPreranked.1623416296068/HALLMARK_HEME_METABOLISM.html]

Details for gene set HALLMARK\_HEME\_METABOLISM[GSEA]

|  || Dataset | state4 |
| Phenotype | NoPhenotypeAvailable |
| Upregulated in class | na\_pos |
| GeneSet | HALLMARK\_HEME\_METABOLISM |
| Enrichment Score (ES) | 0.29047516 |
| Normalized Enrichment Score (NES) | 1.2118007 |
| Nominal p-value | 0.103053436 |
| FDR q-value | 0.23682083 |
| FWER p-Value | 0.874 |
Table: GSEA Results Summary

  

Fig 1: Enrichment plot: HALLMARK\_HEME\_METABOLISM      
 Profile of the Running ES Score & Positions of GeneSet Members on the Rank Ordered List

  

| PROBE | GENE SYMBOL | GENE\_TITLE | RANK IN GENE LIST | RANK METRIC SCORE | RUNNING ES | CORE ENRICHMENT || 1 | BNIP3L |  |  | 54 | 0.294 | 0.0329 | Yes |
| 2 | FOXO3 |  |  | 72 | 0.273 | 0.0688 | Yes |
| 3 | NUDT4 |  |  | 131 | 0.232 | 0.0922 | Yes |
| 4 | PGLS |  |  | 190 | 0.206 | 0.1121 | Yes |
| 5 | SDCBP |  |  | 212 | 0.201 | 0.1371 | Yes |
| 6 | BPGM |  |  | 223 | 0.193 | 0.1628 | Yes |
| 7 | KAT2B |  |  | 347 | 0.166 | 0.1664 | Yes |
| 8 | UROS |  |  | 545 | 0.132 | 0.1531 | Yes |
| 9 | ISCA1 |  |  | 629 | 0.122 | 0.1570 | Yes |
| 10 | PRDX2 |  |  | 634 | 0.122 | 0.1736 | Yes |
| 11 | BTG2 |  |  | 689 | 0.116 | 0.1812 | Yes |
| 12 | ARL2BP |  |  | 759 | 0.108 | 0.1853 | Yes |
| 13 | YPEL5 |  |  | 783 | 0.105 | 0.1965 | Yes |
| 14 | RNF19A |  |  | 793 | 0.104 | 0.2098 | Yes |
| 15 | SEC14L1 |  |  | 817 | 0.102 | 0.2206 | Yes |
| 16 | ADD1 |  |  | 829 | 0.101 | 0.2332 | Yes |
| 17 | TNRC6B |  |  | 833 | 0.101 | 0.2470 | Yes |
| 18 | BLVRB |  |  | 841 | 0.100 | 0.2601 | Yes |
| 19 | SLC6A8 |  |  | 926 | 0.093 | 0.2597 | Yes |
| 20 | EIF2AK1 |  |  | 944 | 0.092 | 0.2700 | Yes |
| 21 | CA2 |  |  | 1037 | 0.085 | 0.2672 | Yes |
| 22 | NARF |  |  | 1136 | 0.079 | 0.2626 | Yes |
| 23 | NCOA4 |  |  | 1176 | 0.077 | 0.2672 | Yes |
| 24 | CAT |  |  | 1179 | 0.077 | 0.2779 | Yes |
| 25 | FBXO7 |  |  | 1239 | 0.074 | 0.2787 | Yes |
| 26 | MXI1 |  |  | 1247 | 0.073 | 0.2880 | Yes |
| 27 | MKRN1 |  |  | 1308 | 0.070 | 0.2883 | Yes |
| 28 | IGSF3 |  |  | 1355 | 0.068 | 0.2905 | Yes |
| 29 | HAGH |  |  | 1580 | 0.059 | 0.2625 | No |
| 30 | HEBP1 |  |  | 1608 | 0.058 | 0.2664 | No |
| 31 | DCAF10 |  |  | 1713 | 0.055 | 0.2573 | No |
| 32 | UCP2 |  |  | 1737 | 0.054 | 0.2613 | No |
| 33 | ALAD |  |  | 1738 | 0.054 | 0.2690 | No |
| 34 | LRP10 |  |  | 1791 | 0.052 | 0.2680 | No |
| 35 | ALDH6A1 |  |  | 1803 | 0.052 | 0.2736 | No |
| 36 | TRAK2 |  |  | 1869 | 0.050 | 0.2702 | No |
| 37 | TMEM9B |  |  | 1918 | 0.049 | 0.2694 | No |
| 38 | LPIN2 |  |  | 1944 | 0.048 | 0.2722 | No |
| 39 | UROD |  |  | 1997 | 0.047 | 0.2704 | No |
| 40 | CIR1 |  |  | 2000 | 0.047 | 0.2767 | No |
| 41 | TCEA1 |  |  | 2132 | 0.043 | 0.2616 | No |
| 42 | ARHGEF12 |  |  | 2202 | 0.042 | 0.2563 | No |
| 43 | OPTN |  |  | 2234 | 0.041 | 0.2571 | No |
| 44 | NR3C1 |  |  | 2265 | 0.040 | 0.2579 | No |
| 45 | XPO7 |  |  | 2465 | 0.036 | 0.2307 | No |
| 46 | ADIPOR1 |  |  | 2543 | 0.034 | 0.2230 | No |
| 47 | SLC25A38 |  |  | 2597 | 0.033 | 0.2191 | No |
| 48 | HTATIP2 |  |  | 2665 | 0.031 | 0.2127 | No |
| 49 | PICALM |  |  | 2677 | 0.031 | 0.2153 | No |
| 50 | CCDC28A |  |  | 2728 | 0.030 | 0.2115 | No |
| 51 | PPP2R5B |  |  | 2925 | 0.026 | 0.1834 | No |
| 52 | MGST3 |  |  | 2993 | 0.025 | 0.1761 | No |
| 53 | RNF123 |  |  | 3018 | 0.025 | 0.1757 | No |
| 54 | PSMD9 |  |  | 3054 | 0.024 | 0.1734 | No |
| 55 | SMOX |  |  | 3127 | 0.023 | 0.1649 | No |
| 56 | DCAF11 |  |  | 3157 | 0.022 | 0.1634 | No |
| 57 | GCLC |  |  | 3344 | 0.017 | 0.1356 | No |
| 58 | VEZF1 |  |  | 3424 | 0.015 | 0.1249 | No |
| 59 | RIOK3 |  |  | 3439 | 0.014 | 0.1247 | No |
| 60 | FBXO9 |  |  | 3491 | 0.012 | 0.1182 | No |
| 61 | GDE1 |  |  | 3549 | 0.011 | 0.1104 | No |
| 62 | UBAC1 |  |  | 3592 | 0.009 | 0.1049 | No |
| 63 | TFDP2 |  |  | 3777 | 0.002 | 0.0753 | No |
| 64 | BCAM |  |  | 3797 | 0.001 | 0.0724 | No |
| 65 | BLVRA |  |  | 3830 | -0.001 | 0.0672 | No |
| 66 | GAPVD1 |  |  | 3895 | -0.003 | 0.0574 | No |
| 67 | AGPAT4 |  |  | 4078 | -0.014 | 0.0299 | No |
| 68 | MARK3 |  |  | 4226 | -0.024 | 0.0094 | No |
| 69 | PIGQ |  |  | 4276 | -0.027 | 0.0053 | No |
| 70 | CAST |  |  | 4301 | -0.029 | 0.0055 | No |
| 71 | SLC11A2 |  |  | 4513 | -0.045 | -0.0223 | No |
| 72 | TFRC |  |  | 4532 | -0.047 | -0.0186 | No |
| 73 | GLRX5 |  |  | 4552 | -0.048 | -0.0149 | No |
| 74 | CCND3 |  |  | 4578 | -0.050 | -0.0119 | No |
| 75 | LAMP2 |  |  | 4579 | -0.050 | -0.0049 | No |
| 76 | RBM5 |  |  | 4613 | -0.052 | -0.0028 | No |
| 77 | CLCN3 |  |  | 4681 | -0.058 | -0.0054 | No |
| 78 | HDGF |  |  | 4737 | -0.063 | -0.0055 | No |
| 79 | RAD23A |  |  | 4743 | -0.063 | 0.0026 | No |
| 80 | NFE2L1 |  |  | 4816 | -0.069 | 0.0007 | No |
| 81 | CTSB |  |  | 4900 | -0.075 | -0.0021 | No |
| 82 | OSBP2 |  |  | 4964 | -0.080 | -0.0010 | No |
| 83 | MBOAT2 |  |  | 4966 | -0.080 | 0.0101 | No |
| 84 | TOP1 |  |  | 5082 | -0.090 | 0.0043 | No |
| 85 | EPB41 |  |  | 5162 | -0.098 | 0.0054 | No |
| 86 | ATP6V0A1 |  |  | 5273 | -0.108 | 0.0028 | No |
| 87 | SLC7A11 |  |  | 5582 | -0.141 | -0.0272 | No |
| 88 | GMPS |  |  | 5613 | -0.146 | -0.0114 | No |
| 89 | SLC25A37 |  |  | 5639 | -0.151 | 0.0059 | No |
| 90 | TSPAN5 |  |  | 5716 | -0.163 | 0.0167 | No |
| 91 | SLC2A1 |  |  | 5995 | -0.237 | 0.0052 | No |
| 92 | BSG |  |  | 6050 | -0.269 | 0.0345 | No |
Table: GSEA details [plain text format]

  

Fig 2: HALLMARK\_HEME\_METABOLISM: Random ES distribution      
 Gene set null distribution of ES for **HALLMARK\_HEME\_METABOLISM**
